# Prevalence and factors associated with the development of asthma in a cohort of children who were preterm infants and received passive immunization with palivizumab

**DOI:** 10.36416/1806-3756/e20260119

**Published:** 2026-11-09

**Authors:** Mariana Bueno Manini, Natali Caroline da Silva, Gabriela do Amaral Gurgel Cruz, André Moreno Morcillo, José Dirceu Ribeiro, Adyléia Aparecida Dalbo Contrera Toro

**Affiliations:** 1. Programa de Pós-Graduação em Saúde da Criança e do Adolescente, Faculdade de Ciências Médicas, Universidade Estadual de Campinas - UNICAMP - Campinas (SP) Brasil.; 2. Centro de Investigação em Pediatria, Faculdade de Ciências Médicas, Universidade Estadual de Campinas - UNICAMP - Campinas (SP) Brasil.; 3. Faculdade de Medicina, Pontifícia Universidade Católica de Campinas - PUCCAMP - Campinas (SP) Brasil.; 4. Departamento de Pediatria, Faculdade de Ciências Médicas, Universidade Estadual de Campinas - UNICAMP - Campinas (SP) Brasil.

**Keywords:** Asthma, Infant, premature, Palivizumab, Risk factors

## Abstract

**Objective:**

To assess the prevalence of asthma in a cohort of preterm infants, comparing those who received prophylaxis with palivizumab, a monoclonal antibody against respiratory syncytial virus (RSV), with those who did not. That is relevant because RSV infection has been linked to recurrent wheezing and asthma in childhood, but a causal relationship remains unclear.

**Methods:**

This was a prospective cohort study involving children who were preterm infants without congenital heart disease, exposed or not to palivizumab. Parents or legal guardians answered a telephone questionnaire adapted from the International Study of Asthma and Allergies in Childhood, which covers respiratory symptoms, allergy history, and potential risk factors.

**Results:**

Among 292 eligible children, 217 were included (149 in the palivizumab group and 68 in the control group). Of those 217 infants, 25.3% (95% CI:19.9-31.5%) had asthma: 24.2% (95% CI: 17.8-31.5%) in the palivizumab group and 27.9% (95% CI: 18.3-39.5%) in the control group, with no significant difference (p = 0.553). In the bivariate analysis, asthma was significantly associated with a family history of atopy (p < 0.001), parental asthma (p = 0.006), allergic rhinitis in the child (p < 0.001), and atopic dermatitis in the child (p = 0.022). In the multivariate analysis, asthma remained associated with a family history of atopy (adjusted OR = 4.16; 95% CI: 1.77-9.79), allergic rhinitis in the child (adjusted OR = 5.10; 95% CI: 2.59-10.02), and parental asthma (adjusted OR = 2.45; 95% CI: 1.19-5.04).

**Conclusions:**

Palivizumab does not seem to protect against asthma development in preterm infants without congenital heart disease. A family history of atopy, parental asthma, and allergic rhinitis in the child seem to be the primary determinants of asthma risk in such infants.

## INTRODUCTION

Asthma is one of the most common chronic respiratory diseases in childhood, with high global prevalence and a significant impact on pediatric morbidity and mortality.^1^ It is recognized as a heterogeneous condition influenced by genetic, environmental, and socioeconomic factors. Allergic sensitization, often accompanied by atopy, increases the risk of respiratory symptoms and contributes to the development of allergic asthma.^2^ Among environmental factors, exposure to tobacco smoke and early respiratory infections, including those caused by respiratory syncytial virus (RSV), are major determinants of asthma development and impaired lung function in childhood.^2^ ^,^ ^3^ In addition, evidence from a birth cohort in Brazil demonstrated that early acute viral bronchiolitis is a significant factor associated with an increased risk of asthma in school-age children.^4^

Palivizumab is a humanized monoclonal antibody used prophylactically to reduce the risk of severe infections caused by RSV in high-risk pediatric populations, including preterm infants with or without bronchopulmonary dysplasia and children with hemodynamically significant congenital heart disease.^5^ Evidence from two double-blind, placebo-controlled clinical trials has demonstrated a significant reduction in RSV-related hospitalizations with its use in these groups.^6^ ^,^ ^7^ However, palivizumab prophylaxis does not provide complete protection against RSV infection. Clinical trials have demonstrated significant, but incomplete, reductions in RSV-related hospitalization among high-risk infants receiving palivizumab prophylactically.^5^ ^,^ ^7^ Therefore, the risk of RSV infection persists even in children who receive the full palivizumab regimen, making it necessary to assess the impact of this partial protection on long-term outcomes.^5^

Several studies have suggested that severe RSV infections during early childhood are associated with an increased risk of developing wheezing and asthma, conditions that may persist into adolescence and early adulthood.^3^ ^,^ ^8^ ^-^ ^12^ However, the causal relationship between RSV infection and the development of asthma remains inconclusive. In one cohort of preterm infants, palivizumab prophylaxis did not reduce the prevalence of atopic asthma in the first six years of life.^13^ Two other, double-blind, placebo-controlled clinical trials also found no association between RSV prophylaxis and subsequent asthma development.^14^ ^,^ ^15^

Beyond RSV infection, genetic and environmental factors interact in a complex manner in the development of childhood asthma.^13^ This interaction is particularly relevant in preterm infants, who exhibit immunologic and pulmonary immaturity, which makes them more vulnerable to severe infections. Although studies such as that conducted by Binns et al.^3^ indicate an association between RSV infection and future asthma risk, uncertainties remain regarding the underlying immunological mechanisms and the effectiveness of interventions such as palivizumab prophylaxis in providing long-term protection against chronic respiratory diseases.^3^ In this context, it is essential to investigate the potential relationship between RSV infection and asthma development, as well as to assess the impact of preventive strategies such as palivizumab, particularly in high-risk populations. Long-term studies are needed in order to elucidate this relationship and evaluate the effect of preventive interventions on asthma development.^2^

The aim of this study was to determine the prevalence of asthma in a cohort of preterm infants without congenital heart disease and to study the impact of palivizumab prophylaxis on asthma development in children 3-13 years of age. We also sought to investigate prenatal and postnatal risk factors associated with asthma development.

## METHODS

This was a prospective cohort study involving preterm infants recruited across two study periods (2011-2012 and 2015-2016). Children were recruited to the palivizumab group during both birth periods, whereas those included in the control group were recruited exclusively during the 2011-2012 birth period. 

For all children who were born prematurely-at a gestational age (GA) < 37 weeks-and received palivizumab prophylaxis during the study periods, the parents or legal guardians were invited to allow that child to participate in the palivizumab group. For eligibility purposes, exposure was defined as receipt of at least two doses of the medication. Children were referred to the Referral Center for Special Biologic Agents at the University of Campinas for passive immunization, originating from pediatrics, neonatology, and pediatric cardiology clinics in cities within Regional Health Division VII of the State of São Paulo, with the University of Campinas as the referral center.

The control group consisted of preterm infants born in 2011 or 2012 with a GA < 37 weeks-as determined by obstetric ultrasound or the Capurro method-at the Comprehensive Women’s Health Care Center of the University of Campinas. Those who did not receive palivizumab were included in the control group.

All preterm infants identified through the Comprehensive Women’s Health Care Center and Referral Center for Special Biologic Agents registries were included. Infants with congenital heart disease were excluded, as were those with any chronic lung disease other than bronchopulmonary dysplasia, pulmonary malformations, cerebral palsy, or other comorbidities that may impair lung function, as well as those who were lost to follow-up.

Formal indications for the use of palivizumab were defined by the Technical Guideline of the São Paulo State Department of Health, published in 2007.^16^ Those indications include having a GA < 28 weeks at birth and being in the first year of life; being a preterm infant of any gestational age with bronchopulmonary dysplasia receiving specific treatment and being ≤ 2 years of age; and having congenital heart disease with hemodynamic repercussions and being ≤ 2 years of age. In addition, Technical Note No. 05/2013 from the Brazilian National Ministry of Health established the guidelines for palivizumab use during the seasonal RSV period, including eligible newborns and hospitalized infants.^17^

Parents or legal guardians were contacted by phone and invited to complete a structured questionnaire. The instrument included questions regarding medical history and items corresponding to a modified version of the International Study of Asthma and Allergies in Childhood (ISAAC) questionnaire, addressing personal information, current allergic respiratory symptoms, and potential exposure factors.^18^ Although originally validated for in specific age groups, the modified ISAAC questionnaire was used in this study only to identify asthma symptoms and prior medical diagnosis, as is commonly done in epidemiological studies involving young children, and did not include instruments designed to assess the level of asthma control. Participants were defined as healthy if they had no medical diagnosis of asthma, no wheezing in the previous 12 months, and no asthma medication in use. To avoid measurement bias, parents or legal guardians completed this questionnaire.

This study was approved by the Research Ethics Committee of the University of Campinas (Reference no. 3.223.902). The content of the informed consent form was read to the parents or legal guardians during the telephone interview, and a copy was subsequently sent via WhatsApp or email. Participation was voluntary, and the decision to withdraw from the study at any time was respected.

Statistical analyses were performed using the Statistical Package for the Social Sciences, version 16.0 for Windows (SPSS Inc., Chicago, IL, USA). Data were recorded on standardized paper forms and subsequently entered into the same software program for analysis.

Quantitative variables were described as mean, standard deviation, quartiles, minimum, maximum, interquartile range, and total range, whereas qualitative variables were presented as absolute and relative frequencies (percentages). The Shapiro-Wilk test was used in order to assess the normality of distributions.

Associations between qualitative variables were evaluated with the chi-square test, Fisher’s exact test, or the Fisher-Freeman-Halton test, as appropriate. The distributions of quantitative variables were compared by using the Mann-Whitney test. The prevalence of asthma and the corresponding 95% confidence intervals were determined with the exact method. 

To evaluate factors associated with asthma, multivariate unconditional logistic regression was applied by using the Wald forward stepwise method, with an inclusion p-value of 0.05 and a removal p-value of 0.06. Variables with p ≤ 0.05 in the bivariate analysis were considered for inclusion, as were those with p-values between 0.05 and 0.20, because of their potential role as confounders. Two logistic regression models were constructed to identify independent predictors of asthma: model 1 included early-life variables, excluding clinical manifestations of atopy in the child, whereas model 2 included those variables, plus clinical data on atopic conditions already present in childhood. The GA and birth weight were included in the multivariate models as continuous variables to preserve statistical power and avoid loss of clinically meaningful information that may occur with categorization. Both variables were treated as potential confounders given their imbalance between the groups. A significance level of 5% (alpha = 0.05) was adopted for all analyses.

## RESULTS

The study initially included 661 records of children who were preterm infants, of whom 409 received palivizumab prophylaxis (palivizumab group) and 252 did not (control group). Of those 661 children, 292 participants were traced and contacted. Of those 292 children, two had died, 25 had other diseases that impair lung function, and 48 had parents or legal guardians who declined to answer the questionnaire. Therefore, there were 217 cases in which the questionnaire was applied: 149 in the palivizumab group and 68 in the control group (Figure 1). Table 1 presents the clinical and demographic characteristics of both groups. The median age at the time of the interview was 7.48 years (range, 3.70-12.18 years) in the palivizumab group and 11.8 years (range, 7.98-13.94 years) in the control group, with a statistically significant difference between the groups (p < 0.001).

**Table 1:** Demographic and clinical characteristics of children who were preterm infants and had received or not received palivizumab prophylaxis (N = 217).

Characteristic	Group		p-value
	Palivizumab	Control	
	(n = 149)	(n = 68)	
Male sex, n (%)	76 (51.0)	41 (60.3)	0.203*
Age at assessment, years, median	7.48	11.80	< 0.001^†^
Mother with higher education completed, n (%)	79 (53.0)	19 (27.9)	0.001*
White race, n (%)	109 (73.2)	43 (63.2)	0.279^‡^
People in the household, n (%)			1.000^‡^
≤ 3	58 (38.9)	26 (38.3)	
≥ 4	91 (61.0)	42 (61.8)	
Family income, n (%)			0.001*
≤ 1 MW	11 (7.4)	16 (23.5)	
1-3× the MW	42 (28.2)	22 (32.4)	
3-5× the MW	30 (20.1)	17 (25.0)	
5-10× the MW	25 (16.8)	7 (10.3)	
> 10× the MW	26 (17.4)	3 (4.4)	
Birth weight, g (median, IQR)	1,100 (1,690)	2,260 (3,585)	< 0.001^†^
Gestational age, weeks (median, IQR)	28 (10)	35 (7)	< 0.001^†^
Breastfeeding duration, months (median, IQR)	3 (44)	8 (48)	0.005^†^
Age at entry into daycare, months (median, IQR)	28.5 (54)	24 (68)	0.192^†^
Bronchopulmonary dysplasia, n (%)	104 (71.7)	0 (0)	< 0.001*
Household pets, n (%)	93 (62.4)	47 (70.1)	0.271*
Exposure to tobacco smoke, n (%)	40 (26.8)	13 (19.4)	0.240*
Family atopy, n (%)	99 (66.4)	49 (72.1)	0.410*
Parental asthma, n (%)	35 (24.1)	19 (27.9)	0.552*

MW: (Brazilian national monthly) minimum wage (1,621 Brazilian reals = US$315 at this writing). *Chi-square test. ^†^Mann-Whitney test. ^‡^Fisher-Freeman-Halton exact test.

**Figure 1:**
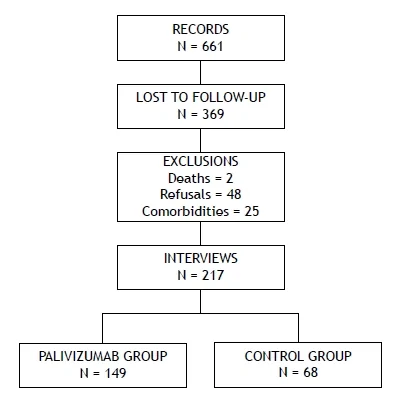
Flow chart of the selection and recruitment process.

The analysis of GA and birth weight showed significant differences between the two groups (p < 0.001 for both variables), indicating that children who were born more prematurely and with lower birth weights were more likely to receive prophylaxis (Table 1). In the palivizumab group, the median GA was 28 weeks (range, 24-34 weeks) and the median birth weight was 1,045 g (range, 510-2,200 g). In the control group, the median GA was 35.0 weeks (range, 29.0-36.0 weeks) and the median birth weight was 2,227.5 g (range, 835-4,420 g).

Bronchopulmonary dysplasia was present in 71.7% of children in the palivizumab group and was not observed in the control group (p < 0.001). Exposure to tobacco smoke was reported for 26.8% of the children in the palivizumab group and 19.4% of those in the control group, with no statistically significant difference between the groups (p = 0.240), as shown in Table 1.

Maternal level of education and household income also differed significantly between the groups. In the palivizumab group, 53.0% of the mothers had completed higher education, compared with 27.9% of those in the control group. Regarding household income, the proportion of families earning less than or equal to the national minimum wage was higher in the control group than in the palivizumab group, whereas the proportion of families earning more than ten times the minimum wage was lower, indicating a higher socioeconomic level among the families of the children in the palivizumab group (Table 1).

Males accounted for 53.9% of the children in the sample as a whole, with no significant difference between the groups. Similarly, no significant differences were found regarding race, mode of delivery, number of people in the household, age at entry into daycare, presence of household pets, exposure to tobacco smoke, family history of atopy, or parental asthma (Table 1).

As shown in Table 2, the overall prevalence of asthma was 25.3% (95% CI: 19.9-31.5). That prevalence was 24.2% (95% CI: 17.8-31.5) in the palivizumab group, compared with 27.9% (95% CI: 18.3-39.5) in the control group. The difference between groups was not statistically significant (p = 0.553).

**Table 2:** Asthma prevalence in children who were preterm infants and had received or not received palivizumab prophylaxis (N = 217).

Group	Prevalence	
	%	95% CI
Palivizumab	24.2	17.8-31.5
Control	27.9	18.3-39.5
Total	25.3	19.9-31.5

In the bivariate analysis, statistically significant associations were observed between asthma and the following variables: family history of atopy (p < 0.001); parental asthma (p = 0.006); allergic rhinitis in the child (p < 0.001); and atopic dermatitis in the child (p = 0.022). Those variables, all related to a personal or family history of atopy, were more prevalent among the children with asthma. Conversely, no differences in asthma prevalence were found in relation to the following (Table 3): sex, race, age of the child, maternal level of education, household income, number of people in the home, mode of delivery, gestational age, birth weight, duration of breastfeeding, age at entry into daycare, pets in the household, exposure to tobacco smoke, or food allergy in the child. Those variables may have acted as confounding factors and were therefore considered in the multivariate analysis.

**Table 3:** Bivariate analysis of factors associated with asthma in children who were preterm infants (N = 217).

Variable	Asthma	Asthma	p-value
	Yes	No	
	(n = 55)	(n = 162)	
Group, n (%)			0.553*
Palivizumab	36 (24.2)	113 (75.8)	
Control	19 (27.9)	49 (72.1)	
Sex, n (%)			0.463*
Female	23 (23.0)	77 (77.0)	
Male	32 (27.4)	85 (72.6)	
Race, n (%)			0.627^†^
White	38 (25.0)	114 (75.0)	
Black	17 (27.9)	44 (72.1)	
Asian	0 (0.0)	4 (100.0)	
Age, years, median (IQR)	9.45 (7.57-11.44)	8.02 (5.81-11.19)	0.183^‡^
Maternal level of education, n (%)			0.070*
Primary education	4 (16.0)	21 (84.0)	
Secondary education	31 (33.0)	63 (67.0)	
Higher education	20 (20.4)	78 (79.6)	
Family income, n (%)			0.302*
≤ 1 MW	8 (29.6)	19 (70.4)	
1-3× the MW	18 (28.1)	46 (71.9)	
3-5× the MW	13 (27.7)	34 (72.3)	
5-10× the MW	5 (15.6)	27 (84.4)	
> 10× the MW	4 (13.8)	25 (86.2)	
Not reported	7 (38.9)	11 (61.1)	
People in household, n (%)			0.065^†^
2	2 (40.0)	3 (60.0)	
3	26 (32.9)	53 (67.1)	
4	17 (20.0)	68 (80.0)	
5	8 (17.8)	37 (82.2)	
6	2 (66.7)	1 (33.3)	
Mode of delivery, n (%)			0.553*
Vaginal	13 (28.9)	32 (71.1)	
Cesarean	42 (24.6)	129 (75.4)	
Gestational age, weeks, median (IQR)	31 (28-34)	29 (28-33)	0.157^‡^
Birth weight, g, n (%)			0.365*
500-1,000	12 (18.5)	53 (81.5)	
1,000-1,500	23 (29.9)	54 (70.1)	
1,500-2,000	6 (21.4)	22 (78.6)	
> 2,000	14 (29.8)	33 (70.2)	
Breastfeeding duration, months, median (IQR)	3 (0-10.5)	4 (1-10)	0.214^‡^
Age at entry into daycare, months, median (IQR)	33 (15-36)	24 (19-36)	0.890^‡^
Pet(s) in the household, n (%)			0.742*
Yes	36 (25.7)	104 (74.3)	
No	19 (23.7)	58 (76.3)	
Exposure to tobacco smoke, n (%)			0.784*
Yes	14 (26.4)	39 (73.6)	
No	40 (24.5)	123 (75.5)	
Family history of atopy, n (%)			< 0.001*
Yes	48 (32.4)	100 (67.6)	
No	7 (10.1)	62 (89.9)	
Parental asthma, n (%)			0.006*
Yes	21 (38.9)	33 (61.1)	
No	32 (20.1)	127 (79.9)	
Rhinitis in the child, n (%)			< 0.001*
Yes	35 (47.9)	38 (52.1)	
No	20 (14.0)	123 (86.0)	
Atopic dermatitis in the child, n (%)			0.022*
Yes	10 (45.5)	12 (54.5)	
No	45 (23.1)	150 (76.9)	
Food allergy in the child, n (%)			0.346*
Yes	6 (31.6)	13 (68.4)	
No	49 (24.9)	148 (75.1)	

MW: (Brazilian national monthly) minimum wage (1,621 Brazilian reals = US$315 at this writing). *Chi-square test. ^†^Fisher-Freeman-Halton exact test. ^‡^Mann-Whitney test.

Table 4 shows the results from the two multivariate unconditional binary logistic regression models. In model 1 (including early-life variables and excluding clinical manifestations of atopy in the child), the only variable that remained significant was a family history of atopy (adjusted OR = 4.16; 95% CI: 1.77-9.79; p = 0.001), indicating that children with a family history of atopy were 4.16 times more likely to develop asthma. In model 2 (including all of the variables analyzed in model 1, plus clinical data on atopic conditions already present in childhood), allergic rhinitis showed a very strong association with asthma development, with a 5.10 times greater likelihood (adjusted OR = 5.10; 95% CI: 2.59-10.02; p < 0.001), followed by parental asthma (adjusted OR = 2.45; 95% CI: 1.19-5.04; p = 0.015).

**Table 4:** Results of the multivariate unconditional binary logistic regression to identify independent predictors of asthma in children who were preterm infants (N = 217).

Analysis	p-value	Adjusted OR	95% CI
Model 1			
Family history of atopy	0.001	4.16	1.77-9.79
Model 2			
Parental asthma	0.015	2.45	1.19-5.04
Allergic rhinitis in the child	< 0.001	5.10	2.59-10.02

Model 1: included early-life variables (family history of atopy, parental asthma, age of the child, maternal level of education, number of household members, and gestational age) but excluded clinical manifestations of atopy in the child; and Model 2: included all of the early-life variables from model 1, plus clinical data on atopic conditions already present in childhood (allergic rhinitis and atopic dermatitis).

## DISCUSSION

To elucidate the role of RSV in the genesis of asthma, we evaluated the prevalence of the disease in children who were preterm infants without congenital heart disease, comparing those who received palivizumab prophylaxis with those who did not. The difference between groups was not statistically significant, suggesting that although palivizumab is effective in preventing severe RSV infections, its use does not substantially modify the genetic or environmental factors involved in the pathogenesis of childhood asthma. These findings indicate that palivizumab should not be considered an isolated strategy for asthma prevention, given that genetic predisposition and environmental exposures play critical roles in the development of the disease.

The scientific literature demonstrates that the prevalence of asthma in preterm children is consistently higher than in the general population, due to pulmonary immaturity and increased susceptibility to early respiratory infections.^2^ ^,^ ^11^ ^,^ ^13^ ^-^ ^15^ Because RSV is a major etiological agent of lower respiratory tract infection in this population, several studies have evaluated whether palivizumab prophylaxis reduces the risk of childhood asthma. However, recent investigations have failed to demonstrate benefits over the long term (6 to 10 years).^2^ ^,^ ^13^ ^-^ ^15^ For example, in a cohort of children born at a GA between 33 and 35 weeks and evaluated at 10 years of age, Kato et al.^2^ reported an asthma prevalence of 16.8% among those who received palivizumab and 19.5% among those who did not. Similarly, Xu et al.,^15^ Mochizuki et al.,^13^ and Scheltema et al.^14^ concluded that immunoprophylaxis had no significant effect on atopic asthma. Conversely, Igde et al.^19^ and Fang et al.^12^ suggested a beneficial association, possibly due to demographic differences among the evaluated populations and distinct methodological approaches. More recently, Hasson et al.^20^ conducted a retrospective cohort study and found no significant effect of palivizumab on long-term asthma outcomes in their multivariate analysis. Similarly, in a systematic review and meta-analysis, Manini et al.^21^ concluded that palivizumab prophylaxis does not reduce asthma risk in children who were preterm infants without congenital heart disease. The findings of the present study align with most of the existing evidence, supporting the idea that palivizumab prophylaxis does not substantially modify the risk of asthma development.

The associations that parental asthma and a family history of atopy show with asthma risk have been widely documented in the literature.^10^ ^,^ ^22^ Robust studies, such as those conducted by Carroll et al.^23^ and Garcia-Garcia et al.,^8^ identified these factors as significant predictors of asthma, results that are corroborated by those of the present study. Our analysis of risk factors revealed statistically significant associations between asthma and a family history of atopy, parental asthma, and allergic rhinitis in the child. The literature consistently demonstrates that genetic factors-particularly the inheritance of genes related to the Th2 immune response-play a central role in asthma pathophysiology.^2^ ^,^ ^13^ ^,^ ^24^ ^,^ ^25^ In addition, the strong association between allergic rhinitis in the child and the subsequent development of asthma underscores the concept that the two conditions share a common inflammatory pathway.^2^ ^,^ ^26^

In the present study, we examined several factors associated with the prevalence of childhood asthma and did not identify significant differences between sexes, although previous studies have suggested that there is a predominance among males.^8^ ^,^ ^27^ Regarding race, no differences were observed between the children who developed asthma and those who did not, which is consistent with the literature indicating that socioeconomic and environmental factors carry greater influence.^28^

Children of mothers with a lower level of education showed a greater predisposition to asthma development. However, a prospective study conducted in Japan suggested that socioeconomic, environmental, and behavioral factors mediate that association.^29^ We did not find asthma to be associated with family income or number of household members, which is consistent with the findings of Jalink et al.^27^ Nevertheless, Unal et al.^22^ reported that more crowded household environments are associated with increased asthma risk in children who were preterm infants.

Perinatal variables such as GA and birth weight were not significantly associated with asthma, although other studies have suggested that a lower GA increases the risk of respiratory morbidities in childhood.^10^ ^,^ ^15^ ^,^ ^27^ ^,^ ^30^ ^-^ ^32^ This may indicate that postnatal lung injury outweighs these early-life risks.^22^ ^,^ ^27^ ^,^ ^32^ A 2022 meta-analysis found that small-for-gestational-age infants did not have an increased risk of asthma.^33^

We found no statistically significant differences between the palivizumab and control groups regarding breastfeeding duration or age at entry into daycare. The literature suggests that these factors interact with specific cofactors.^8^ ^,^ ^10^ ^,^ ^34^ ^-^ ^36^ Although daycare attendance increases the incidence of respiratory infections, there is no evidence that it influences persistent asthma after six years of age.^37^

Exposure to household pets shows variable results in the literature, depending on the family history of asthma and atopy.^34^ ^,^ ^38^ ^,^ ^39^ Prenatal and postnatal exposure to tobacco smoke has been identified as an important risk factor in previous studies, particularly in genetically predisposed children.^2^ ^,^ ^34^ ^,^ ^40^ However, those factors did not show a significant impact in the present analysis. Evidence suggests that the interaction between environmental tobacco smoke and genetic predisposition-such as a family history of asthma-intensifies risk, especially in preterm infants.^22^

Overall, our findings indicate that although palivizumab is effective in preventing severe RSV infection, its use did not have a significant impact on asthma prevalence in the groups studied. This suggests that although immunoprophylaxis remains essential for reducing severe respiratory complications, it does not substantially influence the genetic or environmental determinants associated with asthma. Our multivariate analysis identified a family history of atopy, parental asthma, and allergic rhinitis in the child as independent predictors of asthma risk, underscoring the central roles that genetic predisposition and allergic sensitization play in asthma pathophysiology, which is in line with the current literature. Factors such as a low maternal level of education and exposure to tobacco smoke were not significantly associated with asthma, indicating that other mechanisms may play a more relevant role in the etiology of asthma in the population of children who had been born prematurely.

A large number of children could not be interviewed because they and their families were lost to follow-up. Nevertheless, the consistency of the findings with international cohorts strengthens the external validity of the study.

One strength of the present study is the use of a validated questionnaire, because it enables comparison with international research and enhances the accuracy of respiratory symptom assessment. In addition, the longitudinal follow-up of preterm infants into childhood allowed the assessment of clinically relevant asthma outcomes. To our knowledge, no similar studies have been conducted in Brazil. Despite these strengths, several methodological considerations should be acknowledged when the findings are interpreted. First, exposure to palivizumab was defined as receipt of at least two doses. Although this criterion ensured the inclusion of children who initiated prophylaxis according to the national protocols, some participants may not have completed the full recommended regimen for the RSV season because of clinical eligibility criteria or logistical factors. Consequently, variability in the number of doses may have resulted in heterogeneous levels of protection against RSV infection. Because individual dose records could not be reliably linked to the subgroup of participants successfully recontacted for long-term follow-up, it was not possible to evaluate a potential dose-response relationship or to perform analyses according to prophylaxis completion. Therefore, the impact that variability in the number of doses has on asthma outcomes could not be determined and should be interpreted as a study limitation. In addition, the observational study design introduces the possibility of residual confounding. Although multivariate models were used in order to adjust for relevant clinical and familial variables, unmeasured factors, including bronchiolitis, respiratory hospitalizations during early childhood, and obesity, may still have influenced the observed associations. Variability in asthma diagnostic practices across clinical settings represents an additional limitation. Furthermore, although a structured parent-reported questionnaire adapted from the ISAAC was used to identify physician-diagnosed asthma, diagnostic heterogeneity cannot be entirely excluded. Moreover, the substantial loss to follow-up and reliance on telephone interviews may have introduced selection and recall bias, potentially affecting the representativeness of the study population, although the same methodology was applied consistently to both groups. Important baseline differences between groups, particularly regarding gestational age, birth weight, and bronchopulmonary dysplasia, should also be considered when our findings are interpreted, because those characteristics reflect the clinical criteria for palivizumab eligibility in routine practice.

In conclusion, we have demonstrated that palivizumab prophylaxis in preterm children without congenital heart disease did not have a significant impact on the subsequent development of asthma. A family history of atopy, parental asthma, and allergic rhinitis in the child were identified as the main determinants of asthma risk, highlighting the complex interaction between genetic predisposition and environmental exposure in disease development.

Our findings highlight the need for multifactorial, personalized approaches to asthma prevention, integrating strategies aimed at controlling respiratory infections and atopy. Further research is needed in order to investigate the underlying mechanisms involved in asthma development and to identify highly effective early interventions based on genetic and environmental factors.
